# microRNA: The Impact on Cancer Stemness and Therapeutic Resistance

**DOI:** 10.3390/cells9010008

**Published:** 2019-12-18

**Authors:** Xueqiao Jiao, Xianling Qian, Longyuan Wu, Bo Li, Yi Wang, Xinyu Kong, Lixia Xiong

**Affiliations:** Jiangxi Province Key Laboratory of Tumor Pathogenesis and Molecular Pathology, Department of Pathophysiology, Medical College, Nanchang University, 461 Bayi Road, Nanchang 330006, China; jiaoxueqiao1550@163.com (X.J.); 6302615051@email.ncu.edu.cn (X.Q.); wulongyuan1201@163.com (L.W.); libo181818@126.com (B.L.); 401442719031@email.ncu.edu.cn (Y.W.); xinyu.kong@se17.qmul.ac.uk (X.K.)

**Keywords:** microRNA, cancer, cancer stem cell, therapeutic resistance, cancer therapy

## Abstract

Cancer ranks as the second leading cause of death worldwide, causing a large social and economic burden. However, most anti-cancer treatments face the problems of tumor recurrence and metastasis. Therefore, finding an effective cure for cancer needs to be solved urgently. Recently, the discovery of cancer stem cells (CSCs) provides a new orientation for cancer research and therapy. CSCs share main characteristics with stem cells and are able to generate an entire tumor. Besides, CSCs usually escape from current anti-cancer therapies, which is partly responsible for tumor recurrence and poor prognosis. microRNAs (miRNAs) belong to small noncoding RNA and regulate gene post-transcriptional expression. The dysregulation of miRNAs leads to plenty of diseases, including cancer. The aberrant miRNA expression in CSCs enhances stemness maintenance. In this review, we summarize the role of miRNAs on CSCs in the eight most common cancers, hoping to bridge the research of miRNAs and CSCs with clinical applications. We found that miRNAs can act as tumor promoter or suppressor. The dysregulation of miRNAs enhances cell stemness and contributes to tumor metastasis and therapeutic resistance via the formation of feedback loops and constitutive activation of carcinogenic signaling pathways. More importantly, some miRNAs may be potential targets for diagnosis, prognosis, and cancer treatments.

## 1. Introduction

Cancer is a neoplasm characterized by uncontrolled cell growth and the ability of invasion. It was estimated that in 2018, there were 18.1 million new cancer cases and 9.6 million cancer deaths around the whole word [1]. According to the World Health Organization (WHO), cancer is the second leading cause of death globally, just after cardiovascular diseases [2]. Despite the tremendous effects made in developing first-line anti-cancer drugs, resection surgery, combinational chemotherapy, and radiotherapy, many patients still face high rates of tumor recurrence and metastasis. Currently, most anti-cancer drugs, as well as conventional chemotherapy, mainly target proliferative cancer cells called non-cancer stem cells while the quiescent cancer cells, named cancer stem cells (CSCs), survive [3]. The aggressive characteristics of cancer, such as recurrence, metastasis, and drug resistance, are partially due to the CSCs population [4].

**Figure 1:**
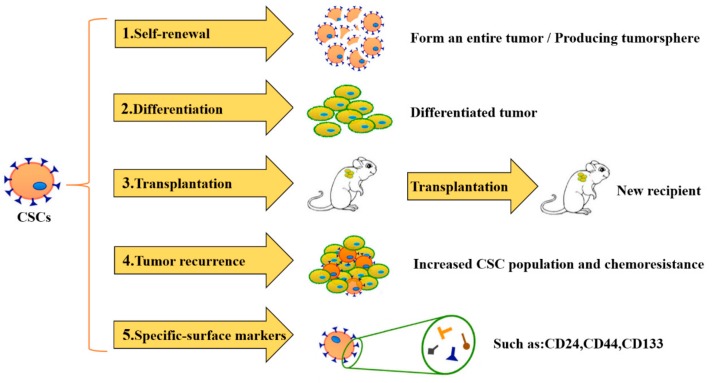
The characteristics of cancer stem cells (CSCs). CSCs have five specific characteristics, which are used for identifying and evaluating cancer stemness. 1. CSCs are able to self-renew and form a tumorsphere. 2. A few CSCs are able to form an entire tumor. 3. CSCs in the recipient can be transplanted to the next recipient. 4. CSCs are responsible for tumor recurrence and chemoresistance. 5. CSCs have many specific stem markers on the cell membrane.

CSCs are pluri- or multi-potent cancer cells, which behave like stem cells in producing heterogeneous cancer cells and have the ability to self-renew [5]. The characteristics of CSCs are known as “cancer stemness” as well, promoting growth of the primary tumor and metastasis of the secondary tumor [6]. CSCs were firstly discovered in the 1890s during an experiment comparing the similarities of tumor tissues with embryonic tissues [6]. It has been proven that a single tumor cell of mice is able to develop into a new tumor in the recipient mouse. During the late 1990s, studies have shown in acute myeloid leukemia cell transplantation that only cells expressing CD34^+^ CD38^−^ form the entire tumor, which are the CSCs of leukemia [7]. Moreover, CSCs were observed to only occupy one per million leukemia tumor cells, indicating that CSCs were identified in leukemia both qualitatively and quantitatively [8]. Then, CSCs were found in a variety of hematopoietic cancers or solid tumors, such as breast, colon, brain, liver, and stomach. Nowadays, it is widely accepted that CSCs are products of combinational genetic and epigenetic changes in normal stem cells. [9]. Besides the stem cell-like properties of proliferation and self-renewal, CSCs have other crucial characteristics as well (Figure 1). Firstly, during CSCs’ proliferation, CSCs form a three-dimensional sphere which is called the tumorsphere. In vitro, the tumorsphere formation assay is widely used for CSCs’ isolation and identification [10]. Secondly, a few CSCs are enough to form an entire tumor [11], as CSCs only account for 1% of all tumor cells and produce heterogeneous tumor cells to form the tumor mass [6]. Thirdly, CSCs in the recipient after transplantation are still able to be transplanted to the next recipient [11]. Fourthly, CSCs are resistant to conventional chemotherapy and radiotherapy [12], which mainly target more mature tumor cells [4], resulting in the enrichment of CSCs in the tumor. The fraction of CSCs positively correlates with tumor progression, metastasis, recurrence, and poor prognosis [13]. Fifthly, CSCs possess distinctive surface membrane markers, which are helpful in CSCs’ identification and isolation [14]. Those markers are proteins on CSCs’ cell membrane, many of which are receptors or antigens. The kinds of CSC markers varies in different tumors; CD24, CD44, and CD133 are three main CSC markers for the most malignant tumors [15]. Due to the ongoing studies and acknowledgement of CSCs’ properties, the significance of developing CSC-targeted cancer therapies in the case of recurrence and metastasis cannot be underestimated.

One of the CSC-targeted therapies eliminates CSCs by targeting related microRNA (miRNA) [16]. miRNAs are about 20- to22-long nucleotides, which belong to the small non-coding RNA family [17]. miRNAs regulate gene expression post-transcriptionally via RNA interference and their levels are inversely associated with the expression of their downstream targets. After cleavage, miRNAs bind to the s3′ untranslated region (3′-UTR) of targeting mRNA, resulting in mRNA silencing [18]. Considering that miRNAs can control multiple gene expressions and the abundance of miRNAs in cells, it is not surprising to find that miRNAs play a role in crucial cell activities, such as cell proliferation, metabolism, and apoptosis. Over 60% of the genes in mammals are regulated by different miRNAs [19]. Most miRNAs are highly evolutionarily conserved, suggesting that miRNAs may be involved in basic biological functions [20]. Abnormal miRNA expression may lead to severe diseases, such as cancer. Studies have shown that abnormal miRNA expression contributes to cancer development in several different ways. miRNAs can be divided into tumor-suppressor and -promoter miRNAs depending on what they regulate and where they are expressed [21]. In recent years, plenty of studies have proven that overexpression or downregulation of various miRNAs in cancers might regulate CSC proliferation and metastasis to facilitate cancer development. In this review, we mainly focus on the relationship between miRNAs and CSCs in the eight most common cancers, hoping to cast new light on miRNA-targeting therapy.

## 2. Lung Cancer

According to histopathological diagnosis, lung cancer can be divided into two main categories. One is small cell lung cancer, accounting for about 15% of lung cancers. The other is non-small cell lung cancer (NSCLC), accounting for about 85% of lung cancers [22]. Except for the conventional therapies, such as anti-tumor drug, chemotherapy, and radiotherapy, accumulating research of lung cancer stem cells has demonstrated that CSC therapy for lung cancer would provide more effective treatment for lung cancer patients.

### 2.1. miR-122

Studies have reported that miR-122 plays a tumor suppressor role by inhibiting the expression of several oncogenes [23,24]. In NSCLC, miR-122 also acts as a tumor suppressor to suppress CSCs in the A549, H460, HCC827 cell line. The induced expression of miR-122 suppresses CSC proliferation by inducing apoptosis, decreasing the stem markers’ expression, as well as sensitizing CSCs to chemotherapy and radiotherapy. miR-122 inhibits the stemness characteristics in NSCLC by binding to peroxiredoxin II (Prx II) mRNA and suppressing its translation. Then, miR-122 improves reactive oxygen species (ROS) intracellular levels to induce apoptosis.

Peroxiredoxins (Prxs) is a superfamily of an ubiquitous cysteine-based anti-oxidant family, and Prxs II is one of the six isoforms of Prx in humans [25]. Prx II contributes to an anti-oxidant effect when it is expressed intracellularly, and has an important function for mediating inflammation when it is expressed extracellularly [26]. Prxs are aberrantly upregulated in several cancers, which has a crucial influence on promoting tumor progression [27].

As the downstream signaling pathway of Prx II, sonic hedgehog (SHH), gil family zinc finger 1 (gli-1) of the Hedgehog pathway, notch receptor 1 (Notch1), hes family bHLH transcription factor 1 (Hes-1) of the Notch pathway, and β-catenin expression are all downregulated after miR-122 induction [28].

### 2.2. miR-128

In lung cancer, miR-128 is identified as a tumor suppressor in A549/paclitaxel (PTX) cell lines. miR-128 inhibits CSC self-renewal, reduces CSC markers expression, and increases the sensitivity to PTX. 

The B-cell-specific Moloney murine leukemia virus insertion site 1 (*BMI-1*) is a proto-oncogene that belongs to the polycomb protein family [29]. BMI-1 plays a role in tumor metastasis through promoting oncogenic transformation and regulating epithelial–mesenchymal transition (EMT) [30]. Therefore, the overexpression of BMI-1 in lung carcinoma indicates aggressive tumor progression and poor prognosis. Mucin 1-C (MUC1-C) is the transmembrane subunit of MUC1 [31]. MUC1-C interacts with tyrosine kinase receptor (TRK), which subsequently activates the MAP kinse-ERK kinase (MEK)/extracellular regulated MAP kinase (ERK) and phosphatidylinositol 3-kinase (PI3K)/AKT serine/threonine kinase 1 (Akt) signaling pathway. MUC1 overexpression is detected in over 80% of NSCLCs, and has a crucial effect on maintaining cancer stemness and clonogenicity [31]. 

miR-128 suppresses BMI-1 and MUC1 expression to reduce stemness protein production and suppresses CSC characteristics in A549/PTX cell lines. Due to the suppression of MUC1-C and BMI-1, the MEK/ERK, PI3K/Akt and Wingless (Wnt)/β-catenin pathways are downregulated, leading to a decrease of the CSC population and impaired chemoresistance to PTX. In short, miR-128 is one of the potential targets for lung cancer treatment [32].

### 2.3. miR-19 

In lung cancer, miR-19 facilitates tumor metastasis by triggering EMT [33]. Moreover, miR-19 promotes tumorsphere formation and increases the expression of stem markers in the A549 and H1299 cell lines of lung cancer.

miR-19 is overexpressed in lung CSCs and it directly suppresses glycogen synthase kinase 3β (GSK3β), which is one of the negative regulators of the Wnt/β-catenin pathway. Therefore, the Wnt/β-catenin pathway is continuously activated in CSCs under the overexpression of miR-19. In short, study of miR-19 may provide new insights for lung cancer treatments [10]. 

### 2.4. miR-410

In NSCLC, miR-410 acts as an oncogenic miRNA and contributes to tumorigenesis. miR-410 facilitates the rate of tumorsphere formation, promotes the expression of stem markers and cell growth, as well as increasing cell resistance to cisplatin in A549 and H1299 cell lines.

miR-410 directly interferes with GSK3β expression. Consequently, the canonical Wnt pathway is hyperactivated in CSCs of NSCLC, which is positively associated with CSC generation maintenance [34,35,36]. In conclusion, miR-410 promotes tumor development and metastasis and enhances stemness via activation of the Wnt/β-catenin pathway [37].

### 2.5. miR-30

miR-30 plays a role as tumor suppressor in the CSCs of NSCLC. Studies show that in the SPC-A1 and NCI-H1650 cell line of NSCLC, miR-30 suppresses tumorsphere formation, inhibits cell growth, and induces cell apoptosis by inhibiting transmembrane-4 L-six family member-1 (TM4SF1).

TM4SF1, a glycoprotein on the plasma membrane, contributes to cell growth and motility [38]. TM4SF1 binds to discoidin domain receptor 1 (DDR1) [39], which is involved in positively regulating the activation of the PI3K/Akt pathway. Due to the downregulation of miR-30, the overexpression of TM4SF1 in CSCs promotes tumorsphere formation and the proliferation of CSCs. In other words, miR-30 interferes with CSC development and induces cell apoptosis [40].

### 2.6. miR-127

miR-127 is vital for lung formation and stem cell differentiation in the embryonic period [41,42]. Overexpression of miR-127 in lung cancer is positively associated with poor prognosis and tumor recurrence. In PC9 and A549 cell lines, oncogenic miR-127 promotes stemness by enhancing self-renewal ability, increasing the expression of stem markers, promoting tumorsphere formation, and increasing cell resistance to gemcitabine via the targeting of tumor necrosis factor alpha-induced 3 (TNFAIP3).

TNFAIP3 is a ubiquitin enzyme, which acts as a negative effector to switch nuclear factor kappa B subunit 1 (NF-κB) signaling off. The overexpression of miR-127 suppresses TNFAIP3 expression, leading to NF-κB pathway activation. The overexpression NF-κB in lung cancer cells, especially CSCs, contributes to cell proliferation, metastasis, and inhibition of apoptosis [43]. Moreover, the upregulation of NF-κB in turn increases miR-127 expression. As a result, miR-127, NF-κB, and TNFAIP3 form a feedforward loop in lung cancer to regulate cell stemness, which promotes CSCs’ self-renewal and accelerates tumorigenesis [44].

The miR-127-mediated positive feedback loop plays a crucial role in tumor aggressiveness, metastasis, and stemness maintenance. Through sustainable self-reinforcing, CSCs enhance the characteristics and enlarge the population. 

### 2.7. miR-129-5p

The downregulation of miR-129-5p is involved in many kinds of tumor development, such as glioblastoma multiforme [45]. In NSCLC cell lines A549 and H460, miR-129-5p reduces CSC markers’ expression, self-renewal ability, and chemoresistance through inhibition of delta-like 1 homolog (DLK1).

DLK1 positively correlates with tumor invasion ability [46]. DLK1 overexpression positively correlates with CSC stemness. By targeting and inhibiting DLK1 expression, miR-129-5p impairs CSCs’ characteristics [47]. The relationship between miR-129-5p and DLK1 helps to reveal the mechanism of the Notch signaling pathway in CSCs.

### 2.8. miR-181b

As a tumor suppressor, miR-181b is downregulated in NSCLC cell lines H1650, H1299, and A549. Additionally, the expression of miR-181b in CSCs decreases stem markers’ expression, suppresses tumorsphere formation, and increases chemosensitivity.

miR-181b is negative regulator of notch receptor 2 (Notch2), which works as an important protein in stem cells. Notch2 is highly expressed in CSCs, promoting the increase of stem markers and the transcription of stem genes. By inhibiting Notch2 translation, miR-181b inhibits stemness characteristics [48]. 

## 3. Breast Cancer

Breast cancer (BC) is one of the most prevalent cancers worldwide, which seriously threatens female health. In 2018, 20.9 million new cases of breast cancer were diagnosed and 6.3 million people died from cancer [1]. Triple-negative breast cancer (TNBC) is the most aggressive breast cancer, with a higher percentage of CSCs [49]. Plenty of studies shown that some miRNAs, such as miR-34a, miR-33b, miR-137, and miR-873, act as tumor suppressors in breast CSCs while other miRNAs, such as miR-221, are overexpressed and act as an oncogene in breast CSCs.

### 3.1. miR-34a

miR-34a targets several different mRNAs to affect breast cancer stemness as a tumor suppressor. The induced overexpression of miR-34a could eliminate some CSCs in breast cancer. miR-34a suppresses tumorshphere formation, CSCs’ self-renewal ability, the expression of stem markers, and drug resistance in the MCF-7 breast cancer cell line by inhibiting serine/threonine-protein kinase D1 (PRKD1). 

PRKD1 overexpression helps to maintain cancer stemness through phosphorylation of protein kinase D/protein kinase C (PKD/PKCμ) and activation of the Wnt/β-catenin pathway [50]. Additionally, phosphorylated PKD/PKCμ has a negative effect on apoptosis through caspase-3 inhibition [51]. As a result, both the number and the self-renewal ability of CSCs are increased. miR-34a-mediated suppression of PRKD1 not only leads to the reduction of stemness and the suppression of tumor growth but also initiates apoptosis, with reduced drug resistance [52]. 

However, the specific mechanism of PRKD1 remains unknown [50], and some research also indicates that PRKD1 has a contrary effect as a tumor suppressor or promoter in different cancer types [53]. For example, PRKD1 is expressed in normal ductal epithelial cells of the breast and inhibits EMT, which is a vital step for tumor cells to acquire the ability of invasion and metastasis [54]. Therefore, the PRKD1 promoter is silenced in invasive breast cancer cells, leading to low expression of PRKD1, which contributes to tumor invasion and metastasis [55]. The discovery of the miR-34a/PRKD1 mechanism may contribute to the investigation of breast cancer and its CSCs on the molecular level. 

Moreover, in the MCF-7 human breast cancer cell line, miR-34a inhibits CSCs’ self-renewal ability, cell proliferation, tumorsphere formation, and the expression of stem markers by suppressing sirtuin1 (SIRT1) expression. 

SIRT1, a nicotinamide adenine dinucleotide-dependent histone deacetylase, plays crucial roles in gene silencing, cell cycle arrest, and apoptosis [56], and it is also involved in maintaining pluripotent stem cells’ generation [57]. In normal conditions, p53, miR-34a, and SIRT1 form a feedback loop to regulate cell apoptosis. p53 expression upregulates miR-34a expression, and miR-34a directly suppresses SIRT1 translation, downregulating SIRT1. SIRT1’s low expression stimulates the acetylation and subsequent activity of p53, which induces cell apoptosis and increases CSCs’ survival rate [58]. 

In breast CSCs, SIRT1 is upregulated while miR-34a is downregulated, leading to an increase of the self-renewal ability of breast CSCs, and the CSC population by suppressing p53-dependent apoptosis [59]. Therefore, by targeting and disturbing the miR-34a–SIRT1 axis in breast CSCs, tumorigenesis could be effectively inhibited, with a lower rate of cancer recurrence and death. 

In addition, in the MCF-7 cell line, miR-34a also decreases CSC stem markers’ expression, suppresses tumorsphere formation, and increases sensitivity to PTX through negatively regulation of Notch1 expression. Notch and its downstream signaling are both involved in the self-renewal and differentiation of breast CSCs [60]. Compared to non-CSCs of breast cancer, CSCs has lower miR-34a expression and higher Notch1 mRNA expression. Both inducing miR-34a and suppressing Notch1 mRNA expression in CSCs would not only lead to the shrinkage of the CSC population and suppressed stemness but also make the tumor mass more sensitive to chemotherapy [61].

### 3.2. miR-185-3p

As a tumor suppressor, the low expression of miR-185-3p in breast cancer always indicates poor prognosis. In the human breast cell line, MDA-MB-468, MDA-MB-231, MDA-MB-453, and MCF-7, miR-185-3p decrease CSC stem markers’ expression, inhibiting cell proliferation, and reducing the quantity and quality of tumorsphere formation.

miR-185-3p directly suppresses E2F transcription factor 1 (E2F1), which is overexpressed in breast CSCs. E2F1 acts as Nanog promoter by enhancing Nanog expression. E2F1 and Nanog overexpression is crucial for CSC properties’ maintenance and tumorigenesis [62]. Therefore, the miR-185-3p/E2F1/Nanog axis is also worthy of studying in miRNA-targeting therapy for breast cancer.

### 3.3. miR-590-5p

miR-590-5p expression is negatively associated with CSCs’ development in the MCF-7 and ZR75-1 cell line. Through inhibition of sex determining region Y (SRY)-box transcription factor 2 (SOX2), miR-590-5p decreases stem marker expression and tumorsphere formation.

SOX2 is a transcription factor (TF), which is abnormally overexpressed in breast CSCs, and responsible for CSCs’ generation and maintenance [63]. SOX2 is vital for embryonic stem cell (ESC) development [64], and its overexpression in breast CSCs contributes to pluripotency maintenance. The abnormal expression of SOX2 in cancers always indicates a higher grade of tumors. 

miR-590-5p directly suppresses SOX2 expression in breast cancer cells. Induced miR-590-5p expression not only decreases the amount of CSCs by through inhibiting SOX2 but also inhibits tumorigenesis significantly [65]. Therefore, by upregulating the expression of miR-590-5p, SOX2 expression is suppressed and tumorigenesis is effectively inhibited.

### 3.4. miR-33b

In the human breast cancer cell lines MCF-7, MDA-MB-231, BT-549, MDA-MB-453, SK-BR-3, and 4T1, miR-33b acts as a tumor suppressor to inhibit CSC properties, such as tumorsphere formation, the expression of stem markers, and self-renewal ability, by inhibiting the high-mobility group AT-hook 2 (HMGA2), spalt-like transcription factor 4 (SALL4), and twist family bHLH transcription factor 1 (Twist1). 

HMGA2, SALL4, and Twist1 are overexpressed in breast cancer, facilitating the self-renewal of CSCs, and enlarging the population of CSCs, leading to the tumor acquiring a higher invasive and metastatic ability [66]. However, it is noticeable that in hematopoietic stem cells, miR-33 can suppress p53 directly to promote stem cells’ self-renewal and inhibit cell apoptosis [67]. Therefore, due to the different effects of miR-33b in CSCs and HSCs, further investigation is warranted for assessing the feasibility of BC therapy by targeting this miRNA.

### 3.5. miR-137

miR-137 inhibits CSC stem markers’ expression, tumorsphere formation, and metastasis in the TNBC cell line MDA-MB-231 and SUM149. miR-137 acts as a tumor suppressor by suppressing B-cell lymphoma/leukemia 11A (BCL11A) expression, leading to the inhibition of CSCs’ self-renewal and proliferation ability, and shrinkage of the tumor mass. 

BCL11A is an oncoprotein, which is highly expressed in breast cancer and involved in maintaining the CSC population. BCLL11A interacts with DNA methyltransferases 1 (DNMT1), which also contributes to CSCs’ maintenance in various cancers, including breast cancer [68]. miR-137 also disturbs the BCL11A–DNMT1 interaction by suppressing BCL11A expression at both the mRNA and protein level [69]. As a result, TNBC development and cancer stemness are impaired. Considering the high chemoresistance and poor prognosis of TNBC patients, using miR-137 to limit the CSC population and inhibit tumorigenesis is quite promising. 

### 3.6. miR-873

miR-873 is a tumor suppressor of breast cancer. In the cell lines MCF-7 and MDA-MB-231, miR-873 is able to reduce the expression of stem markers and pluripotent transcription factors, suppress tumorsphere formation, and lower CSCs’ chemoresistance by inhibiting programmed cell death ligand 1 (PD-L1). 

PD-L1 is an immune checkpoint that is always found as being overexpressed in solid tumors [70]. The higher the expression of PD-L1 in breast cancer, the stronger the cancer stemness is. PD-L1 could propagate the CSC population by continuous hyperactivation of the PI3K/Akt and mitogen activated protein kinase (MAPK)/ERK pathways. 

miR-873 attenuated both the stemness and chemoresistance of BC cells via direct targeting of PD-L1, thus inactivating downstream PI3K/Akt and ERK1/2 signaling. It is notable that compared with the results of Akt/ERK1/2 inhibitors, the results of miR-873/PD-L1 in decreasing the cancer stemness are much more effective, indicating that there must be other pathways involved in the miR-873/PD-L1 axis for CSC generation [71]. 

### 3.7. miR-221

miR-221 acts as a cancer promoter that is overexpressed in breast cancer cells. In the breast cancer cell line T47D, the upregulation of miR-221 promotes tumorsphere formation and the expression of stem markers. miR-221 enhances CSC properties by directly suppressing the expression of DNA methyltransferase 3b (DNMT3b), which could inhibit the expression of stemness genes, such as *Oct3/4* and *Nanog*, by methylating their promoters [72]. miR-221 directly inhibits DNMT3b expression, leading to the overexpression of those stemness genes in CSCs. Therefore, miR-221 overexpression in breast cancer cells usually contributes to an increase of the CSC population and higher probability of recurrence and metastasis [73]. Notably, DNMT3b plays dual roles in different kinds or stages of cancers. Classically, DNMT 3b is recognized as an oncoprotein [74] while in higher tumor stages, it was found that it could act as a tumor suppressor [75]. Thus, whether DNMT3b acts as a tumor suppressor or promoter may depend on the tumor stage and specific tumor type.

## 4. Colorectal Cancer

Colorectal cancer (CRC) is the third most common cancer and the second leading cause of cancer mortality next to lung cancer [1]. Although great progress has been made in CRC clinical therapy, problems, such as chemoresistance and subsequent recurrence, are intractable and need to be settled urgently. CRC is a heterogeneous malignant tumor as well, and as we mentioned before, focusing on the regulatory effect of miRNAs on CSCs may be a potential strategy to solve those problems in CRC treatment.

### 4.1. miR-148a

Low expression of miR-148a in CRCs (e.g., the SW480 cell line) is usually associated with distant metastasis, recurrence, and poor tumor differentiation. As a tumor suppressor, miR-148a not only inhibits stem markers’ expression and tumorsphere formation and reduces drug resistance but also induces apoptosis. 

In CSCs, miR-148a suppresses Wnt family member 10b (Wnt10b) directly. According to a study, Wnt10b plays a crucial role in facilitating tumor progression [76]. Wnt10b aberrant overexpression upregulates the Wnt/β-catenin pathway. Therefore, miR-148a regulates the Wnt/β-catenin pathway negatively to decrease the expression of stem cell-related genes and inhibit CSCs’ self-renewal ability [77]. 

### 4.2. miR-215

miR-215 is a hypoxia-induced miRNA in CRC stem cells. Hypoxia promotes CSCs to form poorly differentiated colonies. miR-215 acts as a tumor suppressor, which withstands the effect of hypoxia on stemness induction. In the CRC cell lines T6, T18, T20, and T51, miR-215 decreases stem markers’ expression, suppresses tumorsphere formation, and reduces CSCs’ self-renewal ability by inhibiting leucine-rich repeating-containing G-protein-coupled receptor (LGR5).

LGR5 is a normal intestinal adult stem cell marker [78], which belongs to the G-protein-coupled receptor family. LGR5 is known as a Wnt agonist receptor that is involved in the Wnt/β-catenin pathway [79]. So, miR-215 decreases the population of CSCs significantly through by the LGR5-activated Wnt/β-catenin pathway [80]. 

### 4.3. miR-195-5p

In the cell lines SW480, SW620, and HT29, miR-195-5p inhibits CSC stem markers’ expression, tumorsphere formation, and reduces cell resistance to 5-FU by inhibiting recombination signal-binding protein of the immunoglobulin kappa J region (RBPJ) and Notch2.

Notch2 is one of the four Notch signal receptors. After binding with Delta ligand, Notch2 undergoes proteolytic cleavage and releases the intracellular domain of Notch (NICD). NICD enters into the nucleus and binds to RBPJ. Consequently, the transcriptions of Notch-targeted genes are activated. The RBPJ-dependent Notch signaling pathway is related with the aggressive CRC tumor phenotype [81]. By negatively regulating the Notch signaling pathway, miR-195-5p decreases the population of CSCs and CRC drug resistance. 

### 4.4. miR-200c

In the CRC cell lines SW480, SW620, HCT116, Lovo, and HT29, the downregulation of miR-200c in CSCs increases tumor sphere formation and self-renewal ability as well as the expression of stem markers. By negatively regulating its downstream target, SOX2, miR-200c inhibits CRC stemness. 

SOX2 plays a crucial role in the self-renewal ability of both normal adult stem cells and CSCs [82]. Notably, SOX2 is not only suppressed by miR-200c, but it also affects the expression of miR-200c in return. According to an experiment, two SOX2 transcription factor-binding sites (TFBSs) are found in the *miR-200c* gene promoter, named TFBS A and B. Studies have shown that it is only when SOX2 binds to TFBS B alone that it can inhibit miR-200c transcription. Normally, SOX2 binds to TFBS A rather than TFBS B. In addition, miR-200c also suppresses the activation of the PI3K/Akt pathway in CSCs, but the inhibitory effect of miR-200c on the PI3K/Akt pathway can be restored by SOX2. The miR-200c/SOX2 feedback loop finally elevates SOX2 expression and promotes CSCs’ characteristics; it should be regarded as a positive feedback loop. However, the reason why the authors recognized it as a negative loop might be that considering miR-200c, it is suppressed by its downstream target. In conclusion, the novel miR-200c/SOX2 negative feedback regulatory loop could be a promising therapeutic target for CRC treatment [83].

### 4.5. miR-30-5p

In the CRC cell lines Caco2, HT29, HCT15, HCT116, SW620, and SW480, miR-30-5p suppresses stem marker expression and tumorsphere formation, inhibits CSC proliferation, and decreases resistance by inhibiting the expression of ubiquitin-specific peptidase 22 (USP22). USP22 is involved in regulating some oncogenic pathway activation [84]. In CRC, because of the low expression of miR-30-5p, USP22 activates the Wnt/β-catenin pathway by increasing the nuclear concentration of β-catenin, and enhancing cancer stemness and tumorigenesis [85].

### 4.6. miR-203

In CRC, miR-203 plays opposing roles in different stages. For example, the serum miR-203 level of stage III–IV patients is higher than that of stage I–II patients [86] In the CRC cell lines HCT-116 and HT-29, miR-203 acts as a tumor suppressor to suppress tumorsphere formation, self-renewal ability, CSC migration, and the expression of stem markers via direct inhibition of GATA-binding protein 6 (GATA6).

GATA6, which belongs to a small family of zinc finger transcription factors, is responsible for normal intestinal epithelium proliferation and maturation [87], CRC’s self-renewal ability, and invasion [88,89]. In CSCs, GATA6 downregulates dickkof-1 (DKK-1), which is a negative effector of the Wnt/β-catenin pathway and upregulates LGR5 to activate the Wnt/β-catenin pathway. 

In short, miR-203 inhibits CRC stemness by suppressing GATA6 and activation of the Wnt/β-catenin pathway, indicating that it might contribute to CRC clinical diagnosis and therapy [90].

### 4.7. miR-139-5p

In the HCT-116 and HT-29 cell lines, miR-139-5p suppress CSCs self-renewal, tumorsphere formation, tumor metastasis, and recurrence as well as stem maker expression via inhibition of transcription factor 4 (TCF4, also known as E2-2).

E2-2 is a basic helix-loop-helix (bHLH) transcription factor of transcription factor 7-like 2 (TCF7L2), which initiates downstream factors of the Wnt/β-catenin pathway. In CRC, the overexpression of E2-2 leads to hyperactivation of the Wnt/β-catenin pathway, contributing to tumor survival and development [91]. Moreover, E2-2 plays a crucial role in promoting EMT [92]. Notably, E2-2 could be stimulated by external factors to regulate the Wnt/β-catenin pathway reversely.

Therefore, by inhibiting E2-2 expression at the protein level, miR-139-5p attenuates CSC stemness, and inhibits tumor metastasis and development [93].

### 4.8. miR-221

In the CRC cell line HCT-116, the overexpression of miR-221 enhances CSCs’ self-renewal and tumorsphere formation ability, increases the expression of stem markers, and suppresses apoptosis by inhibiting Quaking-5 (QKI-5).

QKI-5 is the most abundant isoform of QKI and its presence always indicates good prognosis for patients [94]. Additionally, the reduction of QKI is important for CRC development and the stemness maintenance of both normal stem cells and CSCs [95,96]. Moreover, QKI-5 is involved in EMT regulation as well [97].

miR-221 attenuates the suppressive effect of QKI-5 on CSCs to facilitate enlargement of the CSC population and tumorigenesis. As a result, overexpression of miR-221 usually indicates poor prognosis and a reduced overall survival rate [98]. 

## 5. Prostate Cancer

Prostate cancer (PCa) is the fourth leading cause of cancer incidence and resulted in 3.6 million deaths worldwide in 2018 [1]. Besides surgery to excise the malignant tumor, androgen deprivation therapy (ADT) is the main choice for patients [99]. However, as the tumor progresses, ADT usually fails due to the transition of tumors to castration-resistant prostate cancer (CRPC), which is a serious challenge in PCa therapy [100]. Nowadays, growing evidence shows that EMT and CSCs play a dominant role in PCa metastasis and resistance. That is to say, PCa therapy focusing on EMT and CSCs may broaden our horizon on PCa treatment.

### 5.1. miR-449a 

In the PCa cell lines PC3 and LNCaP, miR-449a decreases stemness markers’ expression and increases CSCs’ chemosensitivity by negatively regulating the expression of prostate leucine zipper (PrLZ), an oncogene that belongs to the tumor protein D52 family.

Studies have shown that PrLZ promotes tumorigenesis and is involved in androgen receptor inactivation in CRPC [101]. In addition, PrLZ interferes with AMPK phosphorylation to protect cancer cells from autography in PCa [102]. Aberrantly high expression of PrLZ increases stem markers’ expression and promotes CSCs’ invasion. 

The main mechanism and function of PrLZ, a newly-discovered PCa-specific oncoprotein, in PCa development deserves further investigations, and the miR-499a/PrLZ axis could be a potential target for eradicating PCa CSCs [103].

### 5.2. miR-7

In the PCa cell line PC3, miR-7 also restrains stem markers’ expression and tumorsphere formation by inhibiting Krüppel-like factor 4 (KLF4).

KLF4 is a zinc-finger transcription factor. In PCa, KLF4 acts as an oncogene and its overexpression activates the PI3K/Akt pathway. In the CSCs of PCa, the upregulation of KLF4 promotes stem cell proliferation and increases the transcription of stem-associated genes. Moreover, as a downstream effector of the PI3K/Akt pathway, phosphorylated p21 in the cytoplasm is responsible for anti-apoptosis while unphosphorylated p21 in the nucleus contributes to G1-S phase arrest [104]. The expression of miR-7 indirectly suppresses the PI3K/Akt pathway, leading to the accumulation of p21 in the nucleus, which mediates the cell cycle arrest of CSCs and impairs stemness. In sum, by inhibiting the KLF4/PI3K/Akt/p21 pathway, miR-7 attenuates cancer stemness, which is sustained for generations, and miR-7 could be a potential marker for PCa prognosis and treatment [105].

## 6. Gastric Cancer

Gastric cancer (GC) is an aggressive tumor with high diagnostic and mortality rates [106], which is listed as one of the top three most malignant tumors [1]. Because of the lack of early significant symptoms and useful diagnostic techniques, patients are usually diagnosed with gastric cancer at late stages with tumor metastasis, facing poor prognosis and low overall survival rates [107]. So, it is urgent that new diagnostic biomarkers and therapeutic strategies are developed. miRNAs have been confirmed to be involved in gastric tumorigenesis and cancer stemness maintenance. 

### 6.1. miR-21

BMI-1 is an oncoprotein whose expression level is upregulated in many types of solid tumors, which is associated with poor prognosis [108]. In the MKN45, SGC-7901, MKN28, and AGS cell lines, BMI-1 upregulates the expression of miR-21, which acts as a tumor promoter and increases stem markers’ expression, tumorsphere formation, and chemoresistance.

p53, phosphatase, and tensin homolog (PTEN) and reversion-inducing cysteine-rich protein with Kazal motifs (RECK) are downstream targets of miR-21 [109,110]. All three are tumor suppressors. In stem cells, the presence of p53 stops cell differentiation and promotes the conversion of stem cells to progenitor cells [111]. PTEN suppresses the PI3K/Akt pathway to inhibit cell differentiation and induce cell death [112]. RECK plays a role in tumor metastasis and angiogenesis by regulating MMPs. By suppressing p53, PTEN, and RECK expression, miR-21 enhances the stemness properties of CSCs and enlarges the CSC population to promote tumorigenesis and metastasis [113]. 

### 6.2. miR-135b

Gastric chronic inflammation increases the tendency of inducing gastric tumorigenesis [114]. Interleukin-1 α/β (IL-1α/β) production in gastritis promotes miR-135b expression, which mediates early and advanced gastric carcinogenesis. The aberrant high expression of miR-135b in SNU-719, SNU-601, SNU-638, and AGS cell lines accelerates the rates of tumorsphere formation, CSC differentiation, and growth.

Forkhead box protein N3 (FOXN3) and RECK are downstream targets of miR-135b. As mentioned before, RECK is an inhibitor of MMPs, which often gets suppressed in carcinogenesis. FOXN3, which is downregulated in several cancers as well, binds to the E2F5 promoter to suppress its subsequent transcription [115,116]. By inhibiting FOXN3 and RECK mRNA expression, miR-135b contributes to early gastric tumorigenesis and enhances stem-like properties. miR-135b, which is involved in primarily gastritis, shows potent oncogenic effects in inducing gastric carcinogenesis. Therefore, miR-135b has the potential to become a prognostic biomarker for early gastric tumor diagnosis [117]. 

### 6.3. miR-577

In the cell lines MKN45 and MGC803, transforming growth factor β (TGF-β) positively regulates the expression of miR-577 via activation of NF-κB. The high expression of miR-577 indicates poor prognosis and a high recurrence rate, and the overexpression of miR-577 stimulates the expression of stem markers and elevates tumorsphere amounts by suppressing the serum deprivation protein response (SDPR).

Studies have shown that SDPR directly inhibits ERK and then inactivates the NF-κB pathway [118]. miR-577 suppress SDPR to attenuate its inhibition of the NF-κB pathway. In consequence, NF-κB in return stimulates the transcription of miR-577, forming a positive feedback loop. 

Considering the crucial role in tumor metastasis and stemness induction, the NF-κB/miR-577/SDPR feedback loop could be a possible target for gastric cancer treatment [119].

## 7. Liver Cancer

Liver cancer is the fifth most common cancer, and is the third cause of cancer mortality [1]. Liver cancer contains two categories: Primary liver cancer and secondary liver cancer. Primary liver cancer is composed of hepatocellular carcinoma (HCC), intrahepatic cholangiocarcinoma, and mixed-type liver cancer. HCC accounts for 90% of primary liver cancers, and a high risk of metastasis as well as recurrence is the main reason why these patients usually have poor prognosis [120]. CSCs are responsible for tumor progression, metastasis, and recurrence. Therefore, focusing on CSCs therapy may provide a new prospect for HCC treatment. 

### 7.1. miR-448

In the HCC Hep3B cell line, miR-448 works as a tumor suppressor to inhibit stem markers’ expression and tumorsphere formation by reducing melanoma-associated antigen 6 (MAGEA6) mRNA expression.

MAGEA is normally expressed in the testis or placenta and plays a crucial role in germ cell development [121]. Aberrantly high MAGEA expression is usually found in invasive malignant tumors, indicating poor prognosis [122]. MAGEA6 suppresses the activation of adenosine monophosphate-activated protein kinase (AMPK), which leads to either cell cycle arrest or apoptosis in HCC [123]. In addition, the overexpression of MAGA6 in CSCs upregulates the expression of stemness markers and pluripotent genes.

miR-448 acts as a tumor suppressor in CSCs by suppressing MAGEA expression and activating the AMPK signaling pathway, leading to attenuated stemness [124]. 

### 7.2. miR-452

In the HepG2, HCC-LM3, and Huh7 cell lines, the upregulation of miR-452 not only promotes stem makers’ expression and tumorsphere formation but also increases chemoresistance and facilitates cell growth by directly inhibiting SOX7.

SOX7, a tumor suppressor, is downregulated in HCC. SOX7 restrains tumor development by interfering with the Wnt/β-catenin signaling pathway [125]. miR-452, which directly suppresses SOX7 expression, is upregulated in the stem cells of HCC and is associated with poor survival rates [126]. 

### 7.3. miR-1305

In the HCC cell lines HCCLM3, HepG2, Hep3B, and Huh7, miR-1305 restrains CSCs’ self-renewal, and the expression of stem markers, and inhibits the tumorsphere quantity by inhibiting the downstream target, ubiquitin-conjugating enzyme E2T (UBE2T).

UBE2T, a member of the ubiquitin-proteasome family, is an oncoprotein in several cancers [127,128]. UBE2T overexpression in HCC contributes to the constitutive activation of the Akt/GSK3β signaling pathway. Therefore, CSCs’ self-renewal ability and the expression of stemness-associated genes are enhanced. miR-1305 directly targets UBE2T mRNA, and mediates UBE2T’s low expression in CSCs, thereby impairing stem cell proliferation and tumorigenesis [129]. For the discovery of the relationship between miR-1305 and UBE2T, the effect of miR-1305 as a prognostic factor in CSC therapy cannot be underestimated.

### 7.4. miR-302a/d

In the cell lines HepG2 and Huh7 of HCC, miR-302a/d suppresses tumorsphere formation and inhibits CSCs’ cell growth by reducing the expression of E2F transcription factor 7 (E2F7).

E2F7 is involved in regulating the cell cycle and its highest expression occurs at the S-phase of the cell cycle [130]. E2F7 is aberrantly overexpressed in the CSCs of HCC, and promotes stem cell proliferation and tumorsphere formation not only through regulating the cell cycle but also activating the Akt/β-catenin/cyclin D1 pathway. 

miR-302a/d binds to the 3’UTR of E2F7 mRNA and inhibits its translation. The induced miR-302a/d expression in CSCs attenuates the stemness through indirect downregulation of the Akt/β-catenin/cyclin D1 pathway, suggesting miR-302a/d is a potential biomarker in HCC stem cell therapy via targeting of E2F7 [131].

### 7.5. miR-217

In the HCC cell lines HepG2 and Huh7, miR-217 acts as oncogenic miRNA to enhance stem cell markers’ expression and promote tumorsphere formation by suppressing the expression of DKK1, a Wnt antagonist, leading to hyperactivation of the Wnt/β-catenin pathway [132].

### 7.6. miR-500a-3p

miR-500a-3p overexpression in HCC is positively associated with poor survival rates. In the HCC cell lines HepG2 and Huh7, miR-500a-3p increases the expression of stem markers and promotes the formation of the tumorsphere. miR-500-3p directly targets and inhibits three negative regulators of the Janus kinase/signal transducer and activator of transcription (JAK/STAT) signaling pathway, suppressor of cytokine signaling 2 (SCOS2), SCOS4, and T-cell protein tyrosine phosphatase non-receptor type (PTPN). 

The JAK/STAT3 pathway contributes significantly to CSCs’ induction and self-renewal [133]. SCOS2 and SCOS4 are two regulators in the negative feedback loop of the JAK/STAT pathway. When SCOS2 and SCOS4 are inhibited, without negative feedback regulation, STAT3 are accumulated in the cytoplasm and nucleus, stimulating pluripotent gene transcription. Studies have shown that high expression of STAT3 in cancer cells positively correlates with tumorigenesis and poor overall survival (OS) [134]. PTPN is an enzyme involved in the removal of phosphate groups from phosphate tyrosine residues to interfere with signal transduction [135]. 

When the three negative regulators are inhibited, miR-500-3p leads to the constitutive activation of the JAK/STAT3 pathway, and CSCs acquire a strong ability for self-renewal, and the population of CSCs is enlarged [136].

### 7.7. miR-612

In the HCC cell lines HCCLM3 and HepG2, miR-612 acts as a tumor suppressor to inhibit tumorsphere formation and stem marker expression by directly inhibiting specificity protein1 (Sp1).

Sp1 is a TF belonging to the KLF family, and Sp1 interacts with the Nanog promoter and facilitates Nanog transcription. Nanog plays a central role in inducing ESCs’ pluripotency [137], and in CSCs, Nanog is upregulated for stemness maintenance. miR-612 directly targets and suppresses Sp1 expression, inhibiting it from binding with the Nanog promoter. Therefore, miR-612 reduces Nanog expression in CSCs. 

Consequently, the expression of miR-612 decreases the amount of CSC stem markers and attenuates CSC-like properties in HCC [138]. miR-612, as a novel biomarker, deserves more study as an miRNA-targeting cancer therapy [139,140,141].

### 7.8. miR-21-3p

In the HepG2 cell line, miR-21-3p inhibits CSC growth and induces cell apoptosis by suppressing methionine adenosyltransferase (MAT) 2A and 2B. 

MAT catalyzes the synthesis of S-adenosylmethionine (SAM). MAT1A and MAT2A are two MAT isoforms: MAT2B encodes an MAT2A regulatory subunit. MAT1A catalyzes more SAM than MAT2A [142]. With the low expression of miR-21-3p, MAT1A expression decreases while MAT2B-mediated MAT2A expression increases in HCC, which is known as “MAT1A:MAT2A switch” [143]. As a result, cells contain a low level of SAM, creating favorable conditions for cell growth [144]. Berberine works as an anti-cancer drug in HCC to induce cell cycle arrest, cell autophagy, and apoptosis [145,146,147]. After HCC cells undergo berberine treatment, miR-21-3p expression increases, leading to CSC growth suppression and apoptosis induction, indicating their therapeutic potential for HCC treatment [148]. 

## 8. Esophagus Cancer

Esophagus cancer ranks as the seventh most common cancer and the sixth highest cancer mortality. In 2018, 5.7 million people were diagnosed with esophagus cancer, and 5.1 million people died from esophagus cancer [1]. Esophageal squamous cell carcinoma (ESCC) is the main histological type of esophagus cancer. Other less common types of esophagus cancer are esophageal adenocarcinoma (EAC) and esophageal small cell carcinoma. The reason why esophagus patients are usually diagnosed at a late stage is the lack of effective biomarkers for diagnosis. At the time of diagnosis, tumors have already spread to other parts of the body, with a high relapse rate [149]. Due to the poor prognosis and low survival rate of esophagus patients, research and exploration of new biomarkers and therapies are urgently required.

### 8.1. miR-17-5p

In the EAC cell lines OE33 P and OE33 R, miR-17-5p is responsible for radiosensitivity and repressing stem-associated gene expression. Chromosome 6 open reading frame 120 (C6orf120) is the downstream target of miR-17, the main function of which is still unknown. C6orf120 expression is elevated in tumor resistance to radiation. Research has shown that C6orf120 could induce primary CD4+ cell apoptosis by mediating intracellular endoplasmic reticulum stress [150].

miR-17-5p suppresses C6orf120 expression to decrease CSCs chemo- and radioresistance. Therefore, miR-17-5p could be a biomarker for CSC resistance evaluation during EAC treatment [151]. 

### 8.2. miR-942

In the ESCC cell lines Kyse510 and Eca109, miR-942 is significantly overexpressed in ESCC, and promotes a higher level of stem markers and pluripotent gene expression. In the clinic, the overexpression of miR-942 is positively correlated with poor prognosis of patients.

miR-942 suppresses the expression of three negative regulators in the Wnt/β-catenin pathway, secreted frizzled-related protein4 (sFRP4), GSK3β, and transducin-like enhancer of split 1 (TLE1). The three negative regulators inhibit Wnt signaling transduction at three different levels. As an extracellular Wnt inhibitor, sFRP4 acts as a Wnt antagonist to inhibit Wnt binding to Frizzled receptors [152]. As an intracellular canonical Wnt pathway inhibitor, GSK3β forms a complex with APC and CK1α to phosphate and degrade β-catenin [153]. As a nuclear suppressive transcription factor, TLE1 interferes with transcription by suppressing the activity of LEF/TCF [154]. 

By suppressing sFRP4, GSK3β, and TLE1, miR-942 activates the canonical Wnt pathway, and consequently promotes the stem-like properties of CSCs and tumorigenesis [155].

### 8.3. miR-21-3p

In the ESCC ECa9706, ECa109, KYSE150, and CAES17 cell lines, upregulation of miR-21-3p promotes the expression of stem markers and pluripotent genes, and resists cell apoptosis via inhibition of the tumor necrosis factor receptor-associated factor4 (TRAF4). TRAF4 interacts with p75^NTR^ and inhibits subsequent NF-κB pathway activation [156]. miR-21-3p suppresses TRAF4 expression, and p75^NTR^ is present in CSCs and activates the NF-κB pathway, restraining cell apoptosis as well as maintaining CSC phenotypes [157].

## 9. Pancreatic Cancer

Pancreatic cancer is one of the most fatal malignant tumors, with 5-year survival lower than 5% [158]. Pancreatic ductal adenocarcinoma (PDAC) is the most common pancreatic cancer, characterized as poorly differentiated, invasive, and with an increase in desmoplasia. The high mortality rate is due, in part, to the difficulties in establishing an early and accurate diagnosis as well as to the low rate of operable resection. Most patients are diagnosed too late to perform surgery, therefore studying and developing miRNA-targeting CSC therapy for pancreatic cancer is an urgent matter of our time.

### 9.1. miR-137

In the pancreatic cancer AsPC-1 and PANC-1 cell lines, miR-137 inhibits the expression of both pluripotent genes and stem markers via suppression of downstream KLF12.

KLF12 is another KLF family member. In PDAC, KLF12 positively regulates Dishelleved 2 (DVL2) at the transcriptional level. DVL2 protects β-catenin from being phosphorylated in the Wnt canonical pathway, therefore KLF12 overexpression in CSCs leads to sustained hyperactivation of the Wnt/β-catenin pathway. Induced miR-137 expression reduces CSC phenotypes. Besides inhibiting stemness, through suppression of KLF12, miR-137 suppresses tumorigenesis as well [159].

### 9.2. miR-1181

miR-1181 is always downregulated in pancreatic cancer, which indicates poor overall survival and a high recurrence rate. In the pancreatic cancer cell lines AsPC-1 and PANC-1, miR-1181 decreases stem markers and pluripotent gene expression, suppresses tumorsphere formation, and shrinks the CSC population by inhibiting SOX2 and STAT3 expression.

SOX2 overexpression in pancreatic cancer contributes to poor differentiation and high invasion [160]. In CSCs, abnormally high SOX2 expression enlarges the CSC population as well [161]. SOX2 also interacts with STAT3 in the JAK/STAT3 pathway, facilitating the transcription of downstream genes. STAT3 is indispensable in normal pancreas development [162], and its overexpression may lead to pancreatic cancer [163]. 

By reducing SOX2 and STAT3 expression, miR-1181 attenuates stemness maintenance and impairs carcinogenesis. SOX2 and STAT3 play crucial roles in promoting tumorigenesis and CSC phenotypes, so that the discovery of miR-1181 may provide a novel target for CSC therapy [164].

### 9.3. miR-30

In the pancreatic cancer cell line Capan-1, miR-30 promotes CSC invasion and migration, and the expression of stemness markers as well as increasing cell resistance. Regulated by CD133, miR-30 facilitates carcinogenesis and maintains cancer stemness. The downstream regulatory pathway of miR-30 in pancreatic CSCs is still unknown. Further investigations should be carried out to understand the effects of miR-30 on pancreatic cancer [165]. 

## 10. The Roles of miRNA Clusters in CSC Pathogenesis

In addition, besides, the dysregulation of single miRNA could be involved in cancer stemness regulation, and some miRNA clusters play vital roles in tumorigenesis, metastasis, and CSC maintenance as well. 

The C19MC miRNA cluster on chromosome 19q13.41 is the largest primate-specific miRNA cluster [166]. miRNAs encoded by C19MC are mainly expressed in placental and germinal tissues [167]. Studies have proven that in embryonal tumors with multilayered rosettes (ETMRs), the amplification of C19MC has oncogenic effects on tumorigenesis and CSC development [168]. Additionally, the gene fusion between the TTYH1 promoter and C19MC results in aberrantly high C19MC expression, which can be detected in brain-specific malignant tumors [169]. C19MC oncomiRNAs work cooperatively to inhibit the expression of tumor suppressors, leading to EMTRs’ rapid proliferation, accelerated cell cycle, and enriched CSC population. In addition, C19MC upregulates MYCN and lin-28 homolog A (LIN28A) by suppressing tip-tap (TTP). LIN28A regulates epigenetic effecters to promote cell growth, survival, and tumor development. C19MC drives tumor cell growth through MYCN-mediated transcriptional regulator circuity, which also shows therapeutic vulnerability [170]. Moreover, C19MC amplification changes DNA methylation by elevating DNMT3b expression, which promotes cell pluripotency and indicates poor prognosis [171,172]. In testicular germ cell tumor (TGCT), the expression of C19MC in advanced tumor stages and non-seminomas (except teratomas) is higher than others [173].

As another miRNA cluster on chromosome 19, the miR-371-373 cluster is deregulated in parathyroid adenomas (PAds), among which the overexpression of miR-372 inhibits the cell cycle regulators, cyclin-dependent kinase inhibitor 1A (p21/CDKN1A) and large tumor suppressor kinase 2 (LATS2), at both the mRNA and protein levels to protect cells from apoptosis. Moreover, miR-372 upregulates parathormone (*PTH*) gene expression as well. Interestingly, miR-372 upregulates DKK1 and downregulates CCND1 thus inhibiting the Wnt/β-catenin pathway, which may explain the limited proliferation ability of PAds [174].

Normally, the miR-17-92 cluster is transcriptionally suppressed by p53 [175]. In lung adenocarcinoma, as nuclear p53 is lost, miR-17-92 is overexpressed during cancer progression [176]. The upregulation of miR-17-92 inhibits the expression of p38α protein, an activator of the Wnt/β-catenin pathway, and the overexpression of leucine-rich repeat-containing G protein-coupled receptor 6 (LGR6). LGR6 is not only an amplifier of the canonical Wnt pathway but a stem marker for CSCs, which enhances CSCs’ self-renewal and differentiation [177]. In CSCs of PDAC, the miR-17-92 cluster promoter is hypermethylated by DNMT1. DNMT1 overexpression contributes to high DNA methylation and CSCs’ self-renewal via suppression of the miR-17-92 cluster [178].

The murine chromosome 12qF1 miRNA cluster is conserved in the human genome on chromosome 14q32 and its expression is increased in lung cancer, indicating poor prognosis. Among the miRNA cluster, miR-494-3p enhances CSCs’ proliferation, stem marker expression, tumor invasiveness, and metastasis by inhibiting PTEN and promoting the NOTCH pathway [179]. In giant cell tumor of the bone (GCT), five miRNAs of chromosome 14q32 are epigenetically silenced or downregulated in the neoplastic stromal cell population (GCTSC), which may be involved in GCT pathogenesis and might play an important role in the malignant transformation of MSCs [180].

Notably, eight miRNAs of the C19MC cluster having an “AAGUGC” sequence as well as members of the miR-302/-372 miRNA cluster have a positive influence on pluripotency induction and stemness maintenance in mesenchymal stem cells. C19MC-AAGUC miRNAs have a hexamer sequence in the canonical seed region, which is effective in targeting gene silencing. This trait may partially explain the reason for reprogramming cell proliferation, inhibiting cell apoptosis, and inducing pluripotency [181]. Additionally, aberrant activation of C19MC is also detected in various cancers, including HCC, brain tumor, and CRC, which plays a crucial role in enhancing CSC characteristics [169,182,183]. In HCC, the presence of C19MC miRNAs indicates a poor prognosis and low survival rate. C19MC promotes poor differentiation of cancer cells’ tumor invasion and CSC development [182,184]. In thyroid adenoma, C19MC and miR-371-3 clusters contribute to tumor development and CSC proliferation and enlargement [185]. In parathyroid tumors, miR-372 is upregulated to inhibit cell apoptosis, and increase PTH synthesis and cell proliferation [174]. C19MC-AAGUC miRNAs and miR-302/-372 are positively involved in tumorigenesis and cancer stemness through complex transcriptional regulatory mechanisms, which deserves continuing investigations and may provide a new prospect for CSC reprogramming and cancer therapy in the near future.

## 11. Discussion and Conclusions

miRNAs, a kind of small non-coding RNA about 22-nucleotide-long, regulates 30% to 50% of all protein-coding genes’ expression at the post-translational level [186]. miRNAs inhibit downstream targets’ expression through RNA interference and silencing. Due to the specificity of various cells, different groups of miRNAs are expressed in different cells and tissues in order to produce specific sets of proteins [187]. Nowadays, thousands of human miRNAs have been identified, each of which are estimated to control hundreds of genes [188]. Therefore, with the ongoing exploration of miRNAs, miRNAs’ clinical application keeps continuously developing as well, among which miRNA-targeting anti-cancer therapy has aroused great interest and wide concern. CSCs are a subpopulation of heterogenous cancer cells. CSCs share significant characteristics with ESCs, especially an unlimited ability to proliferate, self-renew, and differentiate. Moreover, CSCs also acquire resistance to apoptosis and growth inhibitory signals [189]. Those characteristics make sure that CSCs are able to generate heterogenous tumor cells and finally form an entire tumor after metastasis [190]. 

**Figure 2:**
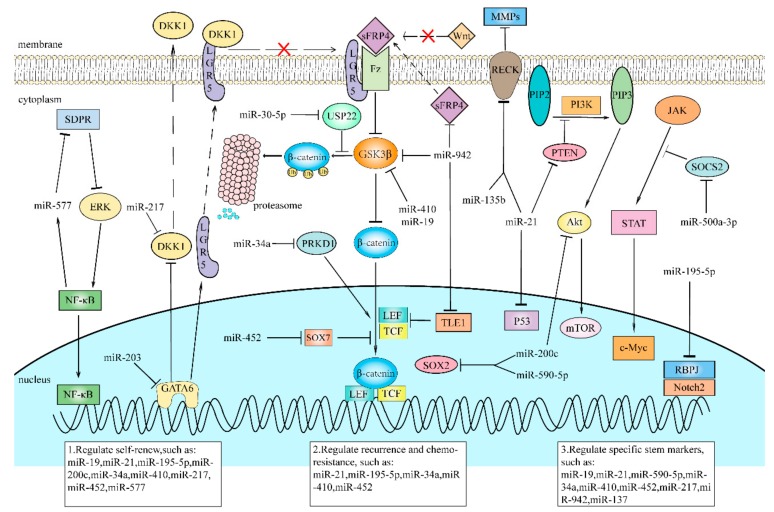
Some miRNAs regulate important pathways to affect CSCs’ maintenance via targeting of their downstream targets.

Plenty of studies and research have shown that the dysregulation of various miRNAs results in the promotion of CSCs properties. We have summarized the roles of various miRNAs in the eight most common cancers (Table 1) and found that these miRNAs play a crucial role in maintaining CSC phenotypes (Table 2). In addition, we selected several miRNAs and summarized their regulation effects on pathways for CSC maintenance (Figure 2). miRNAs in CSCs play a role as either tumor suppressors or promoters, and influences the CSC characteristics and population by regulating downstream genes’ and pathways’ activation (Table 1 and Table 2). In general, as tumor suppressors, they are repressed so that their oncogenic downstream targets are overexpressed, promoting stemness maintenance. As oncogenes, miRNAs are upregulated in CSCs, and their downstream tumor-suppressive targets are suppressed, and as a result, some relative pathways remain constitutively active, which increases the expression of stem-associated genes and enlarges the populations of CSCs.

Lots of studies have paid great attention to the initiated mechanism of CSCs, and two hypotheses have received wide acceptance. One is that due to the accumulation of genetic and epigenetic mutations, normal stem cells have transformed into CSCs, which could explain the reasons for the similar features between normal stem cells and CSCs. Studies have demonstrated that some sets of miRNAs are differently expressed between normal stem cells and CSCs [191]. Using neural stem cells (NSCs) and gliomas cancer stem cells (GSCs) as an example, during NSCs’ differentiation, different groups of miRNAs are expressed orderly to help cells convert into mature neural cells. While in GSCs, miRNAs behave as either tumor suppressors or tumor promoters to maintain GSCs’ stemness and neural cancer hemostasis. Compared with NSCs, 116 microRNAs are overexpressed, and 62 microRNAs are downregulated in GSCs, among which miR-9 and miR-10b are downregulated as tumor suppressors while miR-7 and miR-124 are upregulated as tumor promoters. In human neural progenitor cells (hNPCs), miR-9 is activated for neurosphere formation and differentiation [192,193]. In GSCs, miR-9 is upregulated as an oncomiRNA to facilitate neurosphere formation, and GSC proliferation and invasion [194]. miR-10b is crucial for spatial regulation [195]. miR-10b is a unique tumor promoter in GSCs and is absent in NSCs. High expression of miR-10b leads to uncontrol growth of GSCs [196,197,198]. As a brain-specific miRNA, miR-7 overexpression in NSCs is essential for synapse formation and neurite growth. In GSCs, miR-7 expression is suppressed to promote cell proliferation. Otherwise, miR-7 will induce GSCs’ death by inhibiting several downstream effectors of epidermal growth factor receptor (EGFR) [199]. miR-124 is essential for neural lineage commitment [200,201]. miR-124 is a unique neural miRNA suppressed in GSCs, and miR-124 expression decreases neurosphere formation and stemness markers’ expression, as well as G1 cell cycle arrest [202]. The miRNA network plays a crucial role in the conversion of normal stem cells to CSCs. Changes of miRNA expression may help to distinguish between normal stem cells and CSCs, which has a positive influence on the early diagnosis and treatment of cancer [203].

The other is that differentiated tumor cells could return to CSCs and reacquire stemness properties with changes of the transcriptome. The reversible transformation between CSCs and differentiated tumor cells may better explain the heterogeneity of cancer cells [204]. The transcriptome means the entire set of transcripts in a cell or a certain population of cells. As a part of the transcriptome, long-noncoding RNAs (lncRNAs) are involved in various cellular activities by regulating miRNA expression, mRNA stability and processing, as well as protein stability [205]. For example, in osteosarcoma, lncRNA Fer-1-like protein 4 (FER1L4) inhibits miR18a-5p to induce CSCs apoptosis, and suppress the PI3K/Akt pathway and EMT [206]. In CRC, zinc finger E-box-binding homeobox 2 antisense lncRNA (ZEB2-AS1) directly regulates its mRNA to promote ZEB2 translation [207,208]. In gastric cancer, lncRNA00261 is downregulated because it interacts with slug/GSK3β protein complex, causing slug degradation [209]. Each miRNA could control hundreds or thousands of mRNAs by binding their 3’UTR. Generally, miRNAs always inhibit downstream mRNA expression. However, some miRNAs could promote mRNA expression. For example, pre-miRNAs can induce methylation of gene promoters to stimulate gene expression [210]. RNA-binding proteins (RBPs) occur in every step of RNA processing and the expression of RBPs varies in different tumors [211]. For instance, musashi RNA-binding protein 1 (MSI1) is overexpressed in many kinds of CSCs [212,213,214], and suppresses the expression of NUMB endocytic adaptor protein (NUMB) and JAGGED1, which are two negative regulators of the Notch signaling pathway [215,216]. The epitranscriptome containing all cellular RNA modifications is significant in regulating gene expression. N6-methyladenosine (m^6^A), the most common mRNA modification, plays a fundamental role in promoting CSCs’ self-renewal. It modifies mRNA through a reversible process in which m6A could be removed by “erasers” [217]. In glioblastoma stem cells (GBCs), the removal of methyltransferase-like 3 (Mettl3) and Mettl14 induces a high level of m^6^A mRNA expression, which promotes CSCs’ self-renewal and tumorigenesis [218].

CSCs’ properties, such as self-renewal and therapeutic resistance, are closely related with transcriptional and post-transcriptional mechanisms. Increasing amounts of research focusing on this topic have gradually revealed the mechanisms related to cancer stemness and how they interact with each other. Efforts made on this topic will be of great advantage for us to reach a deep understanding of the initiated mechanism of CSCs, which will cast a new light on future cancer prevention and early diagnosis. Especially, the study of CSCs’ therapeutic resistance will set up a bridge to connect scientific research and clinical therapy, hoping to provide new insights for clinic doctors to work against therapeutic resistance during cancer treatment and elevate prognosis and overall survival for patients. 

Moreover, in some less common cancers, miRNA dysregulation also contributes to carcinogenesis and CSC development as well. In the ovarian cancer cell lines A2780CP20 and SKOV3ip1, miR-21-3p promotes cell proliferation and increases drug resistance by suppressing RNA-binding protein with multiple splicing (RBPMS), POZ domain containing protein 1 (RCBTB1), and zinc finger protein 608 (ZNF 608). Involved in the phosphorylation of SMAD2 and SMAD4 [219], RCBTB1 plays a role in regulating the cell cycle [220]. ZNF608 is associated with transcriptional events [221]. By inhibiting those three downstream targets, miR-21-3p contributes to tumorigenesis and CSC proliferation [222]. In the cervical cancer cell lines Hela, CaSki, Siha, and C33A, miR-23b decreases the tumorsphere amount and size, suppresses stem marker expression, and decreases cell resistance to cisplastin via inhibition of the expression of aldehyde dehydrogenase 1 family member A1 (ALDHA1) [223]. As a marker of tumors, high expression of ALDH1A1 is positively associated with poor prognosis [224]. In the bladder cancer cell lines 5637, BIU87, EJ, SCaBER, and T24, miR-139-5p shrinks the tumorsphere size and amount, and inhibits stem marker and pluripotent gene expression by suppressing BMI-1 [225].

Notably, even the same miRNA may play opposing roles in different tumors. In some tumors, it acts as a suppressor while in other tumors, it acts as a promoter. Take miR-21-3p as an example. The limited and inefficient studies of miR-21-3p show that miR-21-3p plays opposing roles in different cancer tissues. In gastric cancer, the expression of miR-21 facilitates the formation of the tumorsphere and increases the chemoresistance of CSCs by downregulating p53, PTEN, and RECK. In ESCC, miR-21-3p upregulates the expression of stem markers and pluripotent genes, and restrains cell apoptosis by suppressing TRAF4. However, in HCC, miR-21-3p creates an unsuitable environment for CSC growth and induces cell apoptosis by inhibiting MAT2A and MAT2B. It is obvious that miR-21-3p possesses several downstream targets, and in different cancers, miR-21-3p interferes with differing mRNA translation to influence downstream signaling pathway activation. Nevertheless, the specific molecular mechanism of miR-21-3p is still unknown. Another dual-effect miRNA is miR-30. In NSCLC and CRC, miR-30 acts as a tumor suppressor while in pancreatic cancer, miR-30 behaves as a tumor promoter. In NSCLC, miR-30 inhibits cell growth and tumorsphere formation and induces cell apoptosis by negatively targeting TM4SF1, which contributes to PI3K/Akt pathway activation. In CRC, miR-30-5p suppresses stem markers’ expression, tumorsphere formation, and cell proliferation; decreases CSC resistance by inhibiting USP22; and indirectly attenuates activation of the Wnt/β-catenin pathway. However, CD133 upregulates the expression of miR-30 in pancreatic cancer, miR-30 facilitates CSCs’ self-renewal and tumorsphere formation, and increases the expression of stem markers and cell resistance. Downstream targets of miR-30 in pancreatic cancer are still unclear, which deserves further research. miRNAs with opposing effects in different tumors usually regulate multiple pathways’ activation, and studies on those dual-effect miRNAs will cast new light on cancer research and clinical therapy.

As one of the main characteristics of CSCs, therapeutic resistance is the main problem for clinical therapy. Additionally, apoptosis is a significant sign for evaluating therapeutic effect and resistance [226]. Studies have shown that miRNAs play an important role in regulating therapeutic resistance of CSCs. In NSCLC, the low expression of miR-122 promotes Prx II expression and inhibits ROS intracellular levels to reduce cell apoptosis. Besides, the low expression of miR-30 inhibits cell apoptosis by activating TM4SF1. In lung cancer, miR-127 acts as an oncogenic miRNA to inhibit cell apoptosis and increase therapeutic resistance via inhibition of TNFAIP3. TNFAIP3 is a negative regulator of the NF-κB pathway. In BC, miR-873 increases chemo and drug sensitivity via suppression of the immune checkpoint PD-L1 expression. In PCa, PrLZ is related to androgen receptor inactivation, contributing to CRPC. miR-449a elevates CSCs; chemosensitivity by inhibiting PrLZ directly, which provides a new landscape for avoiding CRPC during PCa therapy. In HCC, miR-21-3p suppresses MAT2A and MAT2B to increase SAM expression, impairing hepatoma cell growth. While in ESCC, miR-21-3p promotes cell repair and restrains apoptosis via inhibition of TRAF4 expression. *TRAF4* is a p53-regulated gene and is involved in inducing cell apoptosis as well as suppressing tumorsphere formation [227]. Plenty of research has proven the importance of miRNAs in the therapeutic resistance of CSCs, and some miRNAs might be used as new targets for sensitizing CSCs to clinical treatment via induction of their apoptosis.

**Figure 3:**
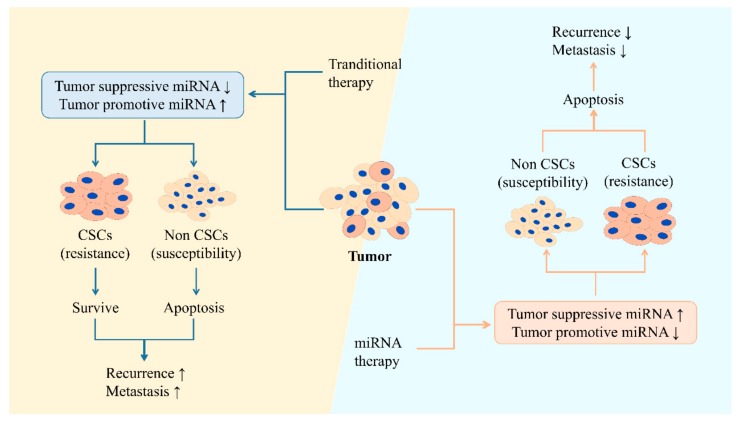
Differences between traditional anti-cancer therapy and miRNA therapy. Traditional therapy mainly kills non-CSCs while CSCs survive with higher therapeutic resistance. By altering the relative miRNA expression in the tumor, miRNA therapy induces CSCs’ apoptosis, lowering the possibility of tumor recurrence and metastasis.

Although huge progression has been made during recent years in the field of cancer treatment, both therapeutic resistance and tumor recurrence are still thorny problems that need to be solved. Current chemotherapy and radiotherapy mainly focuses on killing rapidly dividing cells rather than slow dividing cells; therefore, CSCs evade elimination, with the ability of forming an entire tumor [228]. So, that is the reason of why CSCs are the main reason that resistance and tumor recurrence occurs. Studies have shown that the tumor can be almost eliminated by targeting and destroying CSCs [229,230,231]. CSCs produce plenty of biomolecules, including miRNAs, which have the potential to become valid biomarkers for cancer diagnosis and anticancer therapy. Each miRNA might control hundreds of genes [232], and the dysregulation of miRNAs contributes to the upregulation of pluripotent or multipotent genes as well as stemness factors in CSCs, enhancing stemness phenotypes. That is to say, through altering miRNA expression, complex regulation to suppress CSC proliferation and elimination of the whole tumor may be achieved (Figure 3) [233]. 

Compared with traditional cancer treatments, miRNA-targeting CSC therapy may decrease recurrence and therapeutic resistance, as well as prolong the survival of cancer patients via elimination of both regular cancer cells and CSCs. Moreover, miRNAs have the potential to be developed into effective biomarkers for cancer diagnosis and prognosis. With the progressive understanding and ongoing investigation of CSCs, and new anti-cancer approaches targeting CSCs combined with traditional anti-cancer therapies in the near future, total heterogeneous tumor cells would be removed with little probability of recurrence or therapeutic resistance. However, it is worth noting that miRNA-targeting CSC therapy may be accompanied with some side effects, which deserve attention. For instance, when designing CSCs’ prognostic factors or therapeutic targets, how to distinguish CSCs from normal stem cells or somatic cells and avoid destroying normal stem cells when killing CSCs deserves attention. In conclusion, understanding the specific mechanisms of different miRNAs in maintaining the stemness of different CSCs should benefit us tremendously in the field of cancer research and clinical therapy.

## Figures and Tables

**Table 1:** microRNAs (miRNAs) and their downstream signaling pathways in various cancer stem cells (CSCs).

miRNAs	Cancers	Cell Lines	Suppressor/Promoter	Signaling Pathways	References
miR-122	NSCLC	A549,H460, HCC827	Suppressor	PrxII↓→Hedgehog, Notch, Wnt/β-catenin pathway↓	[28]
miR-128	LC	A549/PTX	Suppressor	BMI-1, MUC1-C↓→PI3K/Akt, MEK/ERK pathway↓	[32]
miR-19	LC	A549, H1299	Promoter	GSK3β↓→Wnt/β-catenin pathway↑	[10]
miR-410	NSCLC	A549, H1299	Promoter	GSK3β↓→Wnt/β-catenin pathway↑	[37]
miR-30	NSCLC	SPC-A1, NCI-H1650	Suppressor	TM4SF1↓→PI3K/AKT pathway↓	[40]
miR-127	LC	PC9, A549	Promoter	TNFAIP3↓→NF-κB pathway↑	[44]
miR-129-5p	NSCLC	A549, H460	Suppressor	DLK1↓	[47]
miR-181b	NSCLC	H1650, H1299, A549	Suppressor	Notch 2↓→Notch pathway↓	[48]
miR-34a	BC	MCF-7	Suppressor	PRKD1↓→GSK3/β-catenin pathway↓	[52]
miR-34a	BC	MCF-7	Suppressor	SIRT1↓→P53 acetylation and activation↑	[59]
miR-34a	BC	MCF-7	Suppressor	Notch1↓→Notch pathway↓	[61]
miR-185-3p	BC	MDA-MB-468, MDA-MB-231, MDA-MB-453, MCF-7	Suppressor	E2F1↓→Nanog↓	[62]
miR-590-5p	BC	MCF-7, ZR75-1	Suppressor	SOX2↓	[65]
miR-33b	BC	MCF-7, MDA-MB-231, BT-549, MDA-MB-453, SK-BR-3, 4T1	Suppressor	HMGA2, SALL4, Twist1↓	[66]
miR-137	TNBC	MDA-MB-231, SUM149	Suppressor	BCL11A↓→BCL11A-DNMT1 interaction↓	[69]
miR-873	BC	MCF-7, MDA-MB-231	Suppressor	PD-L1↓→PI3K/Akt, MAPK/ERK signaling pathway↓	[71]
miR-221	BC	T47D	Promoter	DNMT3b↓→some pluripotent gene expression↑	[73]
miR-148a	CRC	SW480	Suppressor	WNT10b↓→Wnt/β-catenin pathway↓	[77]
miR-215	CRC	T6, T18, T20, T51	Suppressor	LGR5↓→Wnt/β-catenin pathway↓	[80]
miR-195-5p	CRC	SW480, SW620, HT-29, HCT-160	Suppressor	Notch2, RBPJ↓→Notch pathway↓	[81]
miR-200c	CRC	SW480, SW620, HCT116, Lovo, HT29	Suppressor	SOX2, PI3K, Akt↓→PI3K/Akt pathway↓	[83]
miR-30-5p	CRC	Caco2, HT29, HCT15, HCT116, SW620, SW480	Suppressor	USP22↓→β-catenin↓→Wnt/β-catenin pathway↓	[85]
miR-203	CRC	HCT-116, HT-29	Suppressor	GATA6↓→LGR5↓, DKK-1↑→Wnt/β-catenin pathway↓	[90]
miR-139-5p	CRC	HCT-116, HT-29	suppressor	E2-2↓→Wnt/β-catenin pathway↓, EMT↓	[93]
miR-221	CRC	HCT-116	Promoter	QKI-5↓	[98]
miR-449a	PCa	PC3, LNCaP	Suppressor	PrLZ↓→AMPK ↓	[103]
miR-7	PCa	PC3	Suppressor	KLF4↓→PI3K/Akt/p21 pathway↓	[105]
miR-21	GC	MKN45, SGC-7901, MKN28, AGS	Promoter	P53, PTEN, RECK↓→PTEN/Akt pathway ↑	[113]
miR-135b	GC	SNU-719, SNU-601, SNU-638, AGS	Promoter	FOXN3, RECK↓→E2F5 ↑	[117]
miR-577	GC	MKN45, MGC803	Promoter	SDPR↓→NF-κB/miR-577/SDPR axis↑	[119]
miR-448	HCC	Hep3B	Suppressor	MAGEA6↓→AMPK↑	[124]
miR-452	HCC	HepG2, HCC-LM3, Huh7	Promoter	SOX7↓→Wnt/β-catenin pathway↑	[126]
miR-1305	HCC	HCCLM3, HepG2, Hep3B, Huh7	Suppressor	UBE2T↓→Akt/GSK3β pathway↓	[129]
miR-302a/d	HCC	HepG2, Huh7	Suppressor	E2F7↓→AKT/β-catenin/cyclinD1 pathway↓	[131]
miR-217	HCC	HepG2, Huh7	Promoter	DKK1↓→Wnt/β-catenin pathway↑	[132]
miR-500a-3p	HCC	HepG2, Huh7	Promoter	SOCS2, SOCS4, PTPN↓→JAK/STAT3 pathway↑	[136]
miR-612	HCC	HCCLM3, HepG2	Suppressor	SP1↓→SP1/Nanog signaling↓	[138]
miR-21-3p	HCC	HepG2	Suppressor	MAT2A, MAT2B↓→ SAM↓	[148]
miR-17-5p	EAC	OE33 P, OE33 R	Suppressor	C6orf120↓	[151]
miR-942	ESCC	Kyse510, Eca109	Promoter	sFRP4, GSK3β and TLE1↓→Wnt/β-catenin pathway↑	[155]
miR-21-3p	ESCC	ECa9706, ECa109, KYSE150, CAES17	Promoter	TRAF4↓→NF-κB pathway↑	[157]
miR-137	Pancreatic cancer	AsPC-1, PANC-1	Suppressor	KLF12↓→Wnt/β-catenin pathway↓	[159]
miR-1181	Pancreatic cancer	AsPC-1, PANC-1	Suppressor	SOX2, STAT3↓→JAK/STAT3 pathway↓	[164]
miR-30	Pancreatic cancer	Capan-1	Promoter	unknown	[165]

Abbreviations: NSCLC, non-small cell lung cancer; LC, lung cancer; BC, breast cancer; TNBC, triple-negative breast cancer; CRC, colorectal cancer; PCa, prostate cancer; GC, gastric cancer; HCC, hepatocellular carcinoma; EAC, esophageal adenocarcinoma; ESCC, esophageal squamous cell carcinoma; Prx II, peroxiredoxin II; MUC1-C, mucin 1-C; GSK3β, glycogen synthase kinase 3β; TM4SF1, transmembrane-4 L-six family member-1; TNFAIP3, targeting tumor necrosis factor alpha-induced 3; DLK1, delta-like 1 homolog; PRKD1, serine/threonine-protein kinase D1; SIRT1, suppressing sirtuin1; E2F1, E2F transcription factor 1; SOX2, SRY-box transcription factor 2; HMGA2, high mobility group AT-hook 2; SALL4, spalt like transcription factor 4; Twist1, twist family bHLH transcription factor 1; BCL11A, B-cell lymphoma/leukemia 11A; PD-L1, programmed cell death ligand 1; DNMT3b, DNA methyltransferase 3b; LGR5, leucine-rich repeating-containing G-protein coupled receptor; RBPJ, recombination signal binding protein for immunoglobulin kappa J region; USP22, ubiquitin-specific peptidase 22; GATA6, GATA binding protein 6; E2-2, transcription factor4; QKI-5, Quaking-5; PrLZ, prostate leucine zipper; KLF4, Krüppel-like factor 4; PTEN, phosphatase and tensin homolog; SDPR, serum deprivation protein response; MAGEA6, melanoma-associated antigen 6; UBE2T, ubiquitin-conjugating enzyme E2T; E2F7, E2F transcription factor 7; DKK1, dickkof-1; SCOS2, suppressor of cytokine signaling 2; SCOS4, suppressor of cytokine signaling 4; PTPN, T-cell protein tyrosine phosphatase non-receptor type; Sp1, specificity protein1; MAT2A, methionine adenosyltransferase 2A; MAT2B, methionine adenosyltransferase 2B; C6orf120, chromosome 6 open reading frame 120; sFRP4, secreted frizzled-related protein4; TLE1, transducin-like enhancer of split 1; TRAF4, tumor necrosis factor receptor associated factor4; KLF12, Krüppel-like factor 12.

**Table 2:** The effects of miRNAs on CSC phenotypes in various cancers.

miRNAs	Cancers	Cell Lines	Suppressor/Promoter	Effects on Stemness	References
miR-122	NSCLC	A549, H460, HCC827	Suppressor	inhibit I, IV, V	[28]
miR-128	LC	A549/PTX	Suppressor	inhibit I, IV, V	[32]
miR-19	LC	A549, H1299	Promoter	promote I, V	[10]
miR-410	NSCLC	A549, H1299	Promoter	promote I, IV, V	[37]
miR-30	NSCLC	SPC-A1, NCI-H1650	Suppressor	suppress I	[40]
miR-127	LC	PC9, A549	Promoter	promote I, IV, V	[44]
miR-129-5p	NSCLC	A549, H460	Suppressor	suppress I, IV, V	[47]
miR-181b	NSCLC	H1650, H1299, A549	Suppressor	suppress I, IV, V	[48]
miR-34a	BC	MCF-7	Suppressor	suppress I, IV, V	[52]
miR-34a	BC	MCF-7	Suppressor	suppress I, V	[59]
miR-34a	BC	MCF-7	Suppressor	suppress I, IV, V	[61]
miR-185-3p	BC	MDA-MB-468, MDA-MB-231, MDA-MB-453, MCF-7	Suppressor	suppress I, V	[62]
miR-590-5p	BC	MCF-7, ZR75-1	Suppressor	suppress I, V	[65]
miR-33b	BC	MCF-7, MDA-MB-231, BT-549, MDA-MB-453, SK-BR-3, 4T1	Suppressor	suppress I, V	[66]
miR-137	TNBC	MDA-MB-231, SUM149	Suppressor	suppress I, V	[69]
miR-873	BC	MCF-7, MDA-MB-231	Suppressor	suppress I, II, IV, V	[71]
miR-221	BC	T47D	Promoter	promote I, V	[73]
miR-148a	CRC	SW480	Suppressor	suppress I, IV, V	[77]
miR-215	CRC	T6, T18, T20, T51	Suppressor	suppress I, V	[80]
miR-195-5p	CRC	SW480, SW620, HT-29, HCT-160	Suppressor	suppress I, IV, V	[81]
miR-200c	CRC	SW480, SW620, HCT116, Lovo, HT29	Suppressor	suppress I, V	[83]
miR-30-5p	CRC	Caco2, HT29, HCT15, HCT116, SW620, SW480	Suppressor	suppress I, IV, V	[85]
miR-203	CRC	HCT-116, HT-29	Suppressor	Suppress I, V	[90]
miR-139-5p	CRC	HCT-116, HT-29	Suppressor	Suppress I, IV, V	[93]
miR-221	CRC	HCT-116	Promoter	Promote I, V	[98]
miR-449a	PCa	PC-3, LNCaP	Suppressor	suppress IV	[103]
miR-7	PCa	PC3	Suppressor	suppress I, V	[105]
miR-21	GC	MKN45, SGC-7901, MKN28, AGS	Promoter	promote I, IV, V	[113]
miR-135b	GC	SNU-719, SNU-601, SNU-638, AGS	Promoter	promote I, II	[117]
miR-577	GC	MKN45, MGC803	Promoter	promote I, V	[119]
miR-448	HCC	Hep3B	Suppressor	promote I, V	[124]
miR-452	HCC	HepG2, HCC-LM3, Huh7	Promoter	promote I, IV, V	[126]
miR-1305	HCC	HCCLM3, HepG2, Hep3B, Huh7	Suppressor	suppress I, V	[129]
miR-302a/d	HCC	HepG2, Huh7	Suppressor	suppress I	[131]
miR-217	HCC	HepG2, Huh7	Promoter	promote I, V	[132]
miR-500a-3p	HCC	HepG2, Huh7	Promoter	promote I, V	[136]
miR-612	HCC	HCCLM3, HepG2	Suppressor	suppress I, V	[138]
miR-21-3p	HCC	HepG2	suppressor	suppress I	[148]
miR-17-5p	EAC	OE33 P, OE33 R	Suppressor	suppress IV	[151]
miR-942	ESCC	Kyse510, Eca109	Promoter	promote V	[155]
miR-21-3p	ESCC	ECa9706, ECa109, KYSE150, CAES17	Promoter	promote V	[157]
miR-137	Pancreatic cancer	AsPC-1, PANC-1	Suppressor	suppress V	[159]
miR-1181	Pancreatic cancer	AsPC-1, PANC-1	Suppressor	suppress I, V	[164]
miR-30	Pancreatic caner	Capan-1	Promoter	promote, IV, V	[165]

Abbreviations: NSCLC, non-small cell lung cancer; LC, lung cancer; TNBC, triple-negative breast cancer; CRC, colorectal cancer; PCa, prostate cancer; GC, gastric cancer; HCC, hepatocellular carcinoma; EAC, esophageal adenocarcinoma; ESCC, esophageal squamous cell carcinoma. Notes: I. CSCs have self-renew ability and form tumorsphere for proliferation. II. A few CSCs are able to form an entire tumor. III. CSCs in recipient can be transplanted to the next recipient. IV. CSCs are responsible for tumor recurrence and chemoresistance. V. CSCs have many specific stem markers on the cell membrane.
